# Pathological and Practical Researches on the Diseases of the Brain and the Spinal Cord

**Published:** 1835-01-01

**Authors:** 


*Pathological and Practical Researches on the Diseases of the Brain and the Spinal Cord.* By John Abercrombie, M.D., Fellow of the Royal College of Physicians of Edinburgh, &c., First Physician to his Majesty in Scotland. *Third Edition, enlarged.*—Edinburgh and London, 1834. 12mo. pp. 457.

We are by no means inclined *actum agere*, to do what has been already completely done, nor do we wish to imitate the laborious pedestrian in the algebraic problem, who carried his hundred eggs one by one to the basket, ever and anon returning for another, and painfully retracing the same ground. We therefore think it unnecessary to give an abstract, or even a general account, of the admirable work before us. Those of our readers who pursue excellence in the practice of physic with a zeal proportioned to its object must already be well acquainted with Dr. Abercrombie's work on the Brain, and we shall therefore touch on a few points only, confining ourselves entirely, or nearly so, to the new matter inserted in this edition.

For the following remarkable case of *chronic meningitis*, our author declares himself indebted to Mr. Adams, of Banchory.

"Case vii. A gentleman of a cultivated mind, and an amateur painter by profession, about forty-five years of age, had always enjoyed good health, except that latterly he had suffered from ulceration of the tonsils. In the spring of the year 1829, being in London, he felt languid and depressed, owing, as was imagined by himself and his friends, to too ardent application to his professional pursuits; his sight became impaired, his stomach irritable, and he had various other symptoms, which were referred to a morbid derangement of the hepatic system. After being treated for some time upon general principles, he came down to the country, towards the end of the month of June, in expectation that rural retirement would soon restore him to health. During the three succeeding months, the principal symptoms of his complaint were, a torpid state of the bowels, occasional vomiting without nausea, sometimes, though rarely, dull headach, impaired sight, false vision, and ocular spectra. The spectral illusions generally consisted of fantastic female figures dancing around him; and at one time he had the impression of being attended by one of them wherever he went. He was always sensible, however, that they were unreal appearances. He lost strength gradually, his stomach became more and more irritable, and he died on the 12th of October, excessively emaciated.

"*Inspection*. A portion of the dura mater, about three inches by two, immediately to the left of the falx, and a little anterior to its termination in the tentorium, was separated from the skull by a layer of coagulated lymph, imperfectly or not at all organized, and of a dull yellowish red appearance. This portion of the membrane was glued pretty firmly, by the same kind of matter, to the surface of the brain, there being no trace of pia mater or arachnoid visible. This part of the brain was much indurated, especially at one point anteriorly, and to the left, where the induration extended to the depth of an inch and a half; it adhered also to the falx, which was similarly diseased for nearly a square inch throughout its whole thickness; but did not adhere to the right hemisphere of the brain, or involve any part of it in the disease. The brain, in the immediate vicinity of this induration, was somewhat softened; every other part of it was sound, except that there was about a table-spoonful of serum in the ventricles, and as much about the base of the skull. The stomach was not examined.

"This case is a striking example of extensive cerebral disease with very obscure symptoms. It also tends to illustrate the nature of spectral illusions; and it is deserving of remark, as tending to illustrate the shape which these spectral appearances assumed, that about the time this gentleman became ill, his mind was intent upon making a drawing of one of the fanciful descriptions in Moore's "Epicurean," and by the writer of this report it was always supposed that the phantoms which seemed to sport before his eyes bore some resemblance to those which had formerly occupied his imagination.

"It remains to be mentioned, that no circumstance in his life was known to account for the diseased state of his brain, unless that, about three years before his death, he met with a fall upon a stair, whereby he hurt one of his knees seriously, and, as was suspected by his relatives, also sustained some injury of the head. This explanation, however, is merely conjectural." (P. 46.)

Though we are no Broussaists, and are not haunted by the "spectral illusions" of gastro-enteritis, still we must regret that the stomach was not examined: it is probable that no morbid appearance would have been found there, but even this would have been valuable as a piece of negative evidence.

The following case relates to a subject of much interest.

"Case lxxx. A boy aged twelve, the son of a medical friend, had scarlatina mildly in the spring of 1833. Nearly a month after, he was affected with slight anasarca of the face; and, after this had continued several days, he had some vomiting, and appeared languid. About a week after the appearance of the anasarca, he complained one morning of headach, and had some vomiting; pulse slow, and rather languid. About eleven o'clock in the forenoon he suddenly lost his sight, and towards the afternoon he passed into a state bordering on coma. He still complained of headach, but the pulse was not above the natural standard, soft and languid. Topical bleeding having been employed without relief, I saw him at night, and advised general bleeding to ℥xii. to be followed by active purging, and cold applications to the head. During the bleeding the pulse rose both in strength and frequency. Next morning I found him quite sensible, but entirely blind; there was still some headach, but less than formerly, and the pulse was stronger, and not frequent. He was again bled to ℥xii. and the purgatives repeated. After five or six evacuations from the bowels, his sight began to return, and in the evening was entirely restored. Next day he was free from complaint, and has ever since enjoyed good health." (P. 161.)

In speaking of tubercular disease of the cerebellum, our author says,

"I shall only add the following remarkable case, showing in a very striking manner the remissions which take place in the symptoms in diseases of this class, and the periodical character which they sometimes assume.

"Case lxxxv. A gentleman aged thirty-four, in the year 1825 first began to be affected with occasional attacks of headach, which were usually accompanied by vertigo and dimness of sight. In 1827, the pain became more severe, and was distinctly referred to the occiput and superior part of the neck. He had generally remission of it through the day, and aggravations in the evening. In the spring of 1828, the symptoms increased in severity, but he received considerable relief from blistering. In the summer he went to the country, where his general health was much improved, and his headach greatly mitigated. He continued in this improved state till May 1829, when the attacks of headach were again aggravated, accompanied by giddiness, and on one occasion he fell from his chair. In October of the same year, he began to be affected by a most distressing sensation of throbbing, referred to the back part of the head; and he was also affected with vomiting, which continued without intermission for three weeks. The paroxysms of headach were now aggravated to an intense degree of severity. They occurred chiefly in the evening, from six o'clock till midnight, but also at other times of the day. During the more severe attacks, his face was flushed, the vessels on the temples were remarkably distended, and he lay in a state nearly of unconsciousness, unable to speak, and with his hands and arms spasmodically contracted. He still had occasional vomiting and intense acidity of the stomach, and several times complained of double vision: The pulse was generally natural. His situation was now considered as nearly hopeless, and no relief was obtained from any remedies; but, after five or six weeks of intense suffering, the symptoms gradually remitted, and during several weeks in December and January he continued almost free from headach; he was able to walk out, and his general health was greatly improved. In February 1830 the symptoms again increased, but the pain was now chiefly complained of above the eyes; the remissions also were more complete, and upon the whole his sufferings were less severe than during the attack in November. In March, his complaints again subsided, and he was able to take a good deal of exercise in the open air, and to attend a good deal to his business. He had still occasional attacks of headach, but they were not severe, and his condition was considered as much more favorable than it had been for a long time. In the middle of April, the paroxysms of headach became more severe, but by no means in the degree in which they had occurred on former occasions. He was not confined; and no degree of apprehension was excited until the 24th, when in one of those paroxysms he suddenly expired.

"*Inspection.* The ventricles of the brain contained from three to four ounces of limpid fluid, but the surrounding parts were entirely healthy. Imbedded in the substance of the left lobe of the cerebellum there was a tubercular mass, the shape and size of a very small walnut. Externally it was firm, and presented the usual appearance of the scrofulous tubercle; internally it was softened, with the common appearance of unhealthy scrofulous suppuration. The substance of the cerebellum around it was entirely healthy. No other appearance of disease was discovered in the head, and the other viscera were sound." (P. 168.)

In discussing cases of this kind, the point is often mooted, why a constant source of irritation, such as a tubercle in the cerebellum, should allow of any remissions. Perhaps the reason may be, that a paroxysm of pain is not caused until a large quantity of irritability has been accumulated, and that the latter is discharged by the former; an interval of ease succeeding, until a new accumulation has taken place. So that in this, as in many other instances, what is called the disease, is, in fact, merely Nature's method of getting rid of it.

In the third section of the Appendix to Part I. on "certain Affections of the Pericranium," Dr. Abercrombie mentions some cases related by Sir Everard Home, in which "the symptoms in general were headach, with various uneasy feelings in the head, and a painful tenderness of the scalp at a particular spot, with some degree of swelling or thickening of the integuments at the place." (P. 192.) On this subject our author has inserted the following new matter.

"Since the publication of the former edition of this volume, I have seen several cases of this very interesting affection, presenting characters similar to those which I have mentioned in the general description of it, and yielding to a free incision of the part, after the symptoms had been of long continuance, and had resisted much active treatment. One of them, in which the symptoms were very severe, and of eighteen months' standing, has been described by Mr. Blacklock, in the Edinburgh Medical Journal for 1831. In another of the patients, a clergyman, the affection seemed to have been produced by a small piece of plaster which fell on his head from the ceiling of a church. The injury at the time was of the most trifling description; but this affection gradually supervened, accompanied by a train of anomalous nervous symptoms, which greatly impaired his general health, and rendered him entirely unable for his duty for many months. He was quite cured by the incision, which had to be repeated twice.

"In these cases the seat of the disease was distinctly indicated by the tenderness on pressure of a defined spot of the pericranium. But the following case presents some features of great interest, from the complete relief which was afforded by the same operation, though there had been no tenderness at the part, and nothing that led distinctly to the belief that disease of the membrane existed. It has led me to suppose that there are cases of untractable affections of the head in which this treatment might be beneficial, though not distinguished by the symptoms indicating the disease which has led to these observations.

"Case xcvi. A man, aged thirty-nine, upwards of eight years ago, received an injury on his head from the wheel of a waggon. It produced a sore, which healed in about ten days. About eight months after this he began to be affected with attacks of headach, which had continued to recur from that time, though sometimes at long intervals. When I saw him, along with Mr. Kennedy, in autumn 1833, he had been suffering from intense pain in the head for two months. During the whole of this time he had been confined to bed, and unable for any kind of exertion; every kind of active treatment had been employed, without any relief, and the case now exhibited every character of a fixed and formidable disease of the brain. As the principal seat of the pain, he referred to a spot on the vertex, and from this the acute pain seemed to dart into the centre of the brain, and particularly towards the left ear. The spot to which he pointed on the vertex corresponded to that which he represented as the seat of the original wound; but no cicatrix could be discovered, and there was no tenderness of the integuments, nor any other appearance of superficial disease. All the usual remedies, however, having been employed without benefit, I suggested a crucial incision at this part, which was done with complete relief. The wound healed in nine days; he soon after returned to his usual employment as a baker, and through the winter enjoyed good health. During the present summer (1834) he has had a return of headach, after exposure to great heat. After it had resisted various remedies, a repetition of the incision was contemplated, but at present the affection seems to be subsiding." (P. 196.)

Our readers will be interested by another of the new cases.

"*Extravasation in a Cyst, formed by Separation of the Laminæ of the Dura Mater, from Rupture of the middle Meningeal Artery.*

"The following remarkable case was lately communicated by Dr. John Gairdner, to the Medico-Chirurgical Society of Edinburgh, and will appear, in a more detailed form, in the next volume of their Transactions.

"Case cxxi. A man, aged forty-eight, about the 12th of November, 1814, was assisting a neighbour to carry a heavy load to the top of a high stair, when he felt a sudden attack of headach. He was from that time troubled with headach and occasional giddiness, increased by stooping; and, after these symptoms had continued rather more than a fortnight, he became sensible of some imperfection of vision. When seen by Dr. Gairdner, on the 2d of December, he complained of violent headach; pulse forty, and feeble. The pupils were at this time sensible to the light, but after a few days became insensible. He sunk very gradually into coma, without any other remarkable symptom, and died on the 13th.

"*Inspection.* On the left side of the head a cyst was found, in the course of the middle meningeal artery, occupying the region of the lower part of the parietal and upper part of the temporal bone. It was formed by a separation of the laminæ of the dura mater, and contained about four ounces of coagulated blood. The portion of the dura mater forming the cyst was considerably thickened, and very vascular. There was a depression on the surface of the brain corresponding to the cyst, and the ventricles contained a considerable quantity of serous fluid. There was no other morbid appearance." (P. 238.)

Nor will they be dissatisfied with us for extracting the following one.

"A gentleman, aged sixty-four, was first seized with an attack of apoplexy in 1824, from which he recovered under the usual treatment, but retained some imperfection of speech, and a degree of weakness of the left side. Some months after, he had a second attack, and in July 1825 a third, accompanied by convulsions, in which he lay in a state of insensibility for thirty-six hours, and was not able to leave his room for a fortnight. From this time to the period of his death in 1830, he had a succession of apoplectic attacks, amounting in all to twelve. After these attacks, he was generally able to leave his room in a few days, but each left him more and more embarrassed in his speech, and paralytic on the left side, with distortion of the mouth; and he died in the 12th attack in 1830, after an illness of eight or ten days, during which he lay in a state of nearly perfect coma, with total loss of speech, and perfect palsy of the left side.

"*Inspection.* On removing the dura mater, a remarkable depression was found on the surface of the right hemisphere, forming a deep and well-defined cavity, capable of containing from three to four ounces of fluid. It had been filled by a clear serous fluid, which escaped when the dura mater was wounded in opening the head. The surface of the cavity presented nothing different from the ordinary appearance of the cerebral surface, being covered by the pia mater and arachnoid; but the dura mater had been separated by the fluid which had filled the cavity. On cutting into the cerebral substance which formed the cavity, it was found more dense than natural, and a cavity was exposed in the substance of the hemisphere immediately beneath it, presenting the usual appearance of the collapsed cyst, which had been the seat of extravasation. It was about an inch and a half in length, lined by a yellow membrane of the usual appearance, and part of it was obliterated by the adhesion of its opposite surfaces. Several other very small cysts were observed in various parts of the hemisphere, but they were all empty, and no appearance could be discovered capable of accounting for the fatal attack." (P. 267.)

This case is likewise printed in the Edinburgh Medical and Surgical Journal, where Dr. Abercrombie has added the following remarks, which do not appear in the work before us. "The leading peculiarity of this case is the remarkable cavity on the surface of the brain, produced by actual loss of cerebral substance at the part which covered the apoplectic cyst. It presents another fact of some importance, namely, the most distinct approach that I have seen towards an obliteration of the cyst by the adhesion of its opposite surfaces. The French writers describe the cysts as being entirely obliterated in this manner. I was formerly disposed to doubt the accuracy of these observations; but, in this case, about one half of the cyst was obliterated, and there did not appear anything to prevent the obliteration of the remainder." (*Ed. Med. and Surg. Journal,* No. cxxi. p. 255.)

Should any of our readers have lived so far from the centres of medical science as to be unacquainted with this work, we recommend them to procure it immediately; for, to those who wish to be on a level with the knowledge of the age, Dr. Abercrombie's Pathological and Practical Researches are not merely useful, but necessary.

